# Static Permittivity and Electro-Optical Properties of Bi-Component Orthoconic Antiferroelectric Liquid Crystalline Mixtures Targeted for Polymer Stabilized Sensing Systems

**DOI:** 10.3390/polym14050956

**Published:** 2022-02-27

**Authors:** Shantiram Nepal, Banani Das, Malay Kumar Das, Madhumita Das Sarkar, Magdalena Urbańska, Michał Czerwiński

**Affiliations:** 1 Department of Physics, Siliguri Institute of Technology, Siliguri 734009, India; ershantosh22@gmail.com; 2 Department of Physics, University of North Bengal, Siliguri 734013, India; mkdnbu@yahoo.com; 3 Department of Microelectronics and VLSI Technology, Maulana Abul Kalam Azad University of Technology, WB, Kolkata 741249, India; dassarkar.madhumita@gmail.com; 4 Faculty of Advanced Technologies and Chemistry, Military University of Technology, 00-908 Warsaw, Poland; magdalena.urbanska@wat.edu.pl

**Keywords:** antiferroelectric liquid crystals, polymer stabilization, permittivity, spontaneous polarization, response time, rotational viscosity

## Abstract

The behavior of two newly formulated bi-component orthoconic antiferroelectric liquid crystalline (OAFLC) systems, i.e., the Compound A + Compound B mixture system and Compound C + Compound B mixture system has been discussed in light of temperature and concentration dependencies of helical pitch length, spontaneous polarization, relaxation time, bulk viscosity, and the anchoring energy strength coefficient, together with static dielectric permittivity (ε) and dielectric anisotropy. Compound A + Compound B mixtures possess spontaneous polarization between 190–340 nC.cm^−2^ and fast relaxation times between 190–320 µs in the smectic antiferroelectric SmC_A_* phase at room temperature. Compound C + Compound B mixtures also have a spontaneous polarization in the range of 190–280 nC.cm^−2^ and relaxation times in the range of 190–230 µs at room temperature. Most of the mixtures have a helical pitch below one micrometer in the SmC_A_* phase. These advanced mixtures show a broad temperature range of the antiferroelectric SmC_A_* phase, fast switching of molecules under an applied electric field, negative dielectric anisotropy and a short helical pitch, confirming the advantage of designing new polymer-stabilized OAFLC that is targeted for novel application in sensing devices, utilizing the fast responsive electro-optical modulation elements.

## 1. Introduction

The liquid crystalline (LC) state is an intermediate state of matter between the solid and isotropic liquid, which was discovered by an Austrian chemist, Friedrich Reinitzer, in 1888 [1]. Since then, extensive research has been performed in the field of liquid crystals. The potential area of the applicability of LC materials is very broad [2]. For the practical application of LC in electro-optic devices, it is necessary to have a reasonable response time of LC in the order of micro-seconds. Meyer et al. [3] discovered micro-second switching behavior in the ferroelectric SmC* phase of liquid crystal, i.e., the synclinic state, which was experimentally demonstrated by Clark and Lagerwall [4]. However, ferroelectric liquid crystalline materials suffer from reduced brightness due to DC compensation with only one bright state. Later, Chandani et al. [5,6] reported the existence of the antiferroelectric (AF) phase, i.e., the anticlinic state of liquid crystal, formed by chiral rod-like molecules. The chiral ferroelectric (FLC) and antiferroelectric (AFLC) materials reveal definite and very attractive properties: the electro-clinic effect is observed in the orthogonal paraelectric SmA* phase [7,8,9,10], thickness independent memory effect is observed in de Vries’ electro-clinic liquid crystals [11,12,13]. Bistable [14,15,16,17,18,19] and thresholdless [9,20,21,22,23] switching is observed in the synclinic-ferroelectric smectic SmC* phase and electroclinic, bistable, thresholdless and tri-stable switching [24] are observed in the smectic antiferroelectric SmC_A_^*^ phase. In the last few years, the demand for high-switching speed devices has risen tremendously, especially for 3D vision and field-sequential-color (FSC) generation displays [25]. The polymer stabilized blue-phase LCD technology [26] with sub-millisecond switching has also generated huge interest, and there are ongoing activities and development, both in the ferroelectric and antiferroelectric LC areas [27].

AFLC’s possess tri-stable switching behavior, which gives sharp thresholds, easy DC compensation and micro-second responses, and has intrinsic analog gray scale capability, with no ghost effect and the possibility of passive driving [5,6,28]. However, it has experienced a poor contrast due to a strong influence from poor homogeneity of AFLC bookshelf alignment and the so-called pre-transitional effect [29,30,31,32]. Orthoconic AFLC materials (OAFLCs) [33,34,35,36] were proposed as an alternative, as they possess high-speed switching, high-contrast and a dark state, which is ideally perfect [28]. Although OAFLCs are proper materials for achieving lossless phase modulation in three-level phase-only modulator [37] and in beam-steering applications [38], it is necessary to mention that there is a large asymmetry between switching times (τ_on_ and τ_off_), which is even much larger than that for nematic materials or other non-orthoconic smectic materials. This effect is closely related to a delicate balance between the synclinic-ferroelectric (F) and anticlinic-antiferroelectric (AF) state, which in turn strongly depends on the molecular structure of the LC materials, as well as the polarity and surface structure of the cell. This delays the relaxation process, rendering the F-state metastable [39]. The problem of the metastable F-state in AFLCs is solved by a polymer stabilization of the orthoconic state. A small amount, typically <5% weight, of reactive LC monomer and dimmer, and a suitable photo-initiator are mixed into the OAFLC before filling the cell. The orthoconic state is then illuminated by ultraviolet light to cross-link the reactive monomer. After polymer stabilization, the F-state is not metastable any longer, and a rapid relaxation from the bright to dark state is ensured by switching off the field [39,40,41]. Furthermore, V-shaped electro-optic switching [42,43,44] is also a promising effect for fast switching device applications. The polymer-stabilized OAFLC materials possessing a short helical pitch are also particularly appealing due to their potential application in effects based on the deformation of the helical structure [45]; they have attracted increasing interest, mainly due to their potential applications for flow, pressure, gas and electric smart sensing systems used in many significant fields [46,47,48,49,50,51], and therefore, the development of a liquid crystal base mixture is crucial. For all the above-mentioned sensing applications, one of the key points is to develop suitable bi-component OAFLC mixtures to further polymerize them by using suitable monomers and dimers to produce materials with appropriate features. In this work, two binary systems have been prepared to achieve the targeted liquid crystalline properties [52,53,54,55,56,57,58] by tuning the parameters, such as mesomorphic behavior, and electro-optic, structural, dielectric and helical properties according to the specific application requirements. A limited number of antiferroelectric mixtures [59,60,61,62], with either a very long or very short helical pitch, as well as the continuous development of principally new applications of liquid crystalline materials, makes the formulation of new antiferroelectric mixtures with desired helical parameters a significant step in this direction. Therefore, the aim of this work is to prepare and investigate the main properties of the two bi-component OAFLC mixture systems. The sequence of mesophases, the temperature and concentration dependences of the helical pitch, spontaneous polarization, switching time, as well as the viscosity anchoring energy coefficient and static permittivity have been studied to identify those mixtures with suitable and optimal electro-optic properties for further doping with reactive mesogens and a subsequent forming of a polymer network to stabilize and symmetrise the targeted antiferroelectric phase.

## 2. Materials and Methods

Bi-component mixtures were formulated from three pure antiferroelectric compounds. Sequence of mesophases and the transition temperature of the three pure compounds are as follows: (a) Crystal < 30 °C SmC_A_* 81.5 °C Iso for Compound A, (b) Crystal < 30 °C SmC_A_* 93 °C SmC* 108 °C SmA* 110 °C Iso for Compound B and (c) Crystal < 30 °C SmC_A_* 93 °C SmC* 109 °C SmA* 111 °C Iso for Compound C taken during the cooling cycle. The details on the design and synthesis of these pure compounds are presented in Refs. [63,64,65].


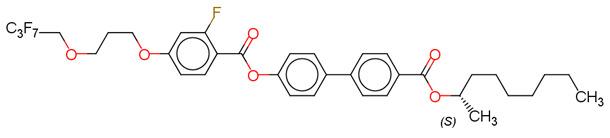
 (S)-4′-(1-methyloctyloxycarbonyl)biphenyl-4-yl4-[3-(2,2,3,3,4,4,4-heptafluorobutoxy)prop-1-oxy]-2-fluorobenzoate (Compound A) [63]


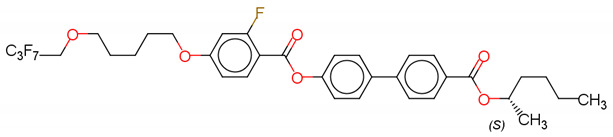
(S)-4′-(1-methylpentyloxycarbonyl)biphenyl-4-yl4-[5-(2,2,3,3,4,4,4-heptafluorobutoxy) pentyl-1-oxy]-2-fluorobenzoate (Compound B) [63]


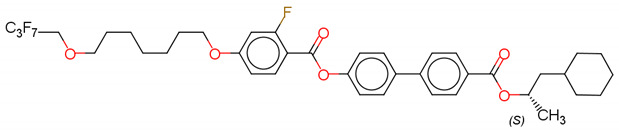
(S)-4′-(1-methylcyclohexyloxycarbonyl)biphenyl-4-yl4-[7-(2,2,3,3,4,4,4-heptafluorobutoxy)heptyl-1-oxy]benzoate (Compound C) [63]

Two binary mixtures were prepared: (i) the Compound A + Compound B mixture system with mole fraction: x = 0.1, 0.2, 0.3, 0.4, 0.5, 0.6, 0.7, 0.8, 0.9 (x is the concentration of Compound A in the mixture) and (ii) the Compound C + Compound B mixture system with mole fraction: x = 0.1, 0.2, 0.4, 0.6, 0.8 (x is the concentration of Compound C in the mixture). The pure LC compounds were taken in a vial with a fixed proportion (mole fraction) after weighting the liquid crystal material using a high precision digital balance (Mettler Toledo AB-265-S). The compounds were mixed thoroughly by placing the vial in an ultra-sonicator (SONAPROS PR 250-MP) for several hours at a fixed temperature above their clearing temperatures. These mixtures were filled in homogeneously aligned (HG) commercially available LC cells (AWAT Poland) made up of indium tin oxide (ITO) coated glass plates with a cell gap of ~5 µm thickness and an active area of 0.25 cm^2^, using capillary force and the mixtures were allowed to slowly cool down to room temperature.

Phase transitions temperatures were determined by a polarizing optical microscope (POM) (Motic BA300, 5MP resolutions). The uncertainty in the measurement of phase transition temperatures from thermal scanning is 0.1 °C. The helical pitch length was measured by the selective light reflection method where the intensity of the transmitted light was measured by a Shimadzu UV-VIS-NIR spectrometer in the range of 360–3000 nm. The AML WU7 temperature controller with a Peltier element was used with an accuracy of 0.1 °C, the details of this procedure are given in Ref. [66]. Electro-optical measurements have been performed by applying the square wave of voltage V_pp_ = 36 V, f = 20 Hz to the sample filled cells by the Picotest (G5100A) arbitrary waveform generator and FLC voltage amplifier (F20A). The applied field inverts the polarization of the polar LC molecules and a current response was observed. The current response was recorded by a digital storage oscilloscope (1052A), suitably interfaced with a computer, the detailed method of which is given in Ref. [67]. The relaxation time, τ, rotational viscosity, η, and the anchoring coefficients W_p_ and W_d_ were evaluated for all the mixtures, by a method, the description of which has been given by us in our earlier publications [67]. Static dielectric measurement was performed using a digital LCR-bridge (Agilent E4980A); the permittivity parallel and perpendicular to the molecular long axis, ε_||_ and ε_⊥_, respectively, and hence the dielectric anisotropy (Δε = ε_||_ − ε_⊥_) were estimated by measuring the capacitance of a liquid crystal cell. The perpendicular component of the electric permittivity (ε_⊥_) was measured in the planar configuration (homogeneous, HG cell) with 0.5 V external voltage. The parallel component of the permittivity (ε_||_) was measured by applying an external voltage of 20 V in the HG cell to align the samples to homeotropic configuration. Both parallel and perpendicular components of the dielectric permittivity were measured at a constant frequency of 1 kHz. Further details of the determination of the static permittivity are given in Refs. [68,69].

## 3. Results

### 3.1. Mesomorphic Behavior

**Figure 1:**
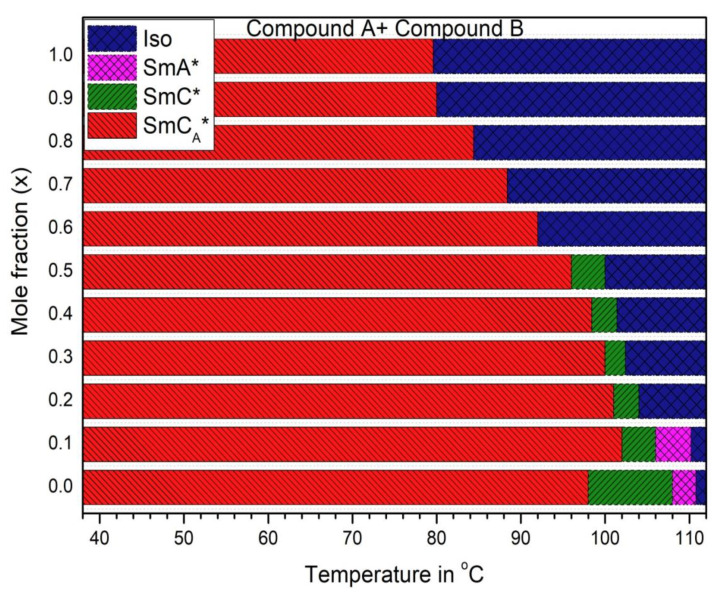
Sequence of mesophases and phase transition temperatures of the Compound A + Compound B mixture system with mole fraction: x = 0.0, 0.1, 0.2, 0.3, 0.4, 0.5, 0.6, 0.7, 0.8, 0.9, 1.0 (x is the molar concentration of Compound A in the mixture) recording using POM during cooling.

Phase transition temperatures and sequence of mesophases for the mixtures were obtained by carefully observing the characteristic textures and their changes in POM; this was cross-verified by the optical transmission method. Three types of phase sequences were detected: (i) Cr-SmC_A_*-SmC*-SmA*-Iso; (ii) Cr-SmC_A_*-SmC*-Iso; (iii) Cr-SmC_A_*-Iso. The mixtures possess chiral anticlinic SmC_A_* phase in a wide temperature range, the ferroelectric tilted SmC* phase in a much smaller range, and the paraelectric orthogonal SmA* phase but only in a quite short temperature range at high temperatures just before the isotropic (Iso) phase transition. Figure 1 presents the phase transition temperatures of the Compound A + Compound B mixture system with mole fraction: x = 0.0, 0.1, 0.2, 0.3, 0.4, 0.5, 0.6, 0.7, 0.8, 0.9, 1.0 (x is the molar concentration of Compound A in the mixture).

Compound A (x = 1.0) possesses the direct SmC_A_*-Iso phase transition at 81.5 °C. With the decrease in molar concentration of Compound A in the mixture, the temperature of the direct SmC_A_*-Iso phase transition increases by about ~11.5 °C to reach 92 °C (at x = 0.6). On further decrease in the mole fraction of Compound A, the SmC* phase starts to be more favorable. Mixtures with a direct SmC_A_*-Iso phase transition may be used as a component of mixtures in OAFLC materials to resolve the occurrence of chevron defects usually present in conventional materials possessing the Cr-SmC_A_*-SmC*-SmA*-Iso phase transitions; thus, these mixtures may be utilized as potential smart materials for high-contrast, 3D displays applications.

**Figure 2:**
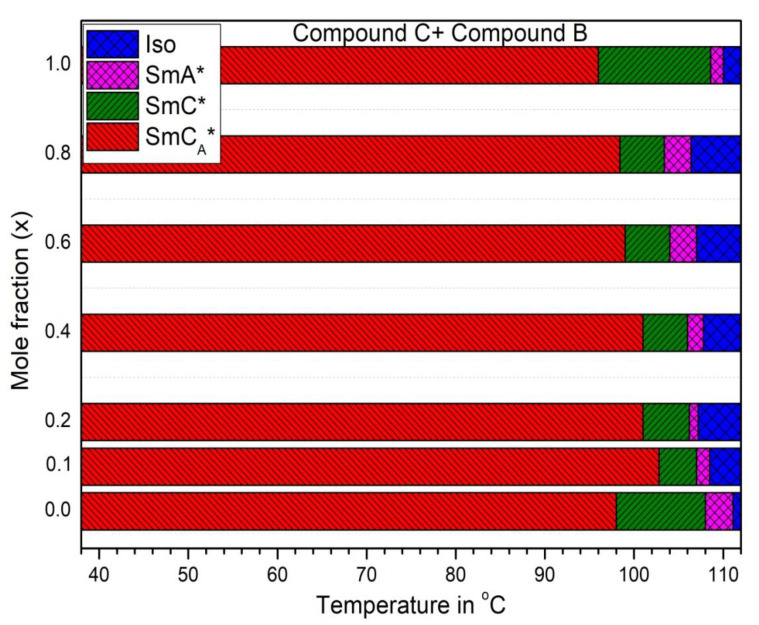
Sequence of mesophases and phase transition temperatures of the Compound C + Compound B mixture system with mole fraction: x = 0.0, 0.1, 0.2, 0.4, 0.6, 0.8, 1.0 (x is the concentration of Compound C in the mixture) recording using POM during cooling.

The sequence of mesophases and the phase transition temperatures for the Compound C + Compound B mixture system is shown in Figure 2 (Mole fraction x = 0.0, 0.1, 0.2, 0.4, 0.6, 0.8, 1.0 corresponds to the concentration of Compound C in the mixture). It is clearly observed that it is possible to effectively tune and increase the temperature range of the SmC_A_* phase in the resulting mixture system.

**Figure 3:**
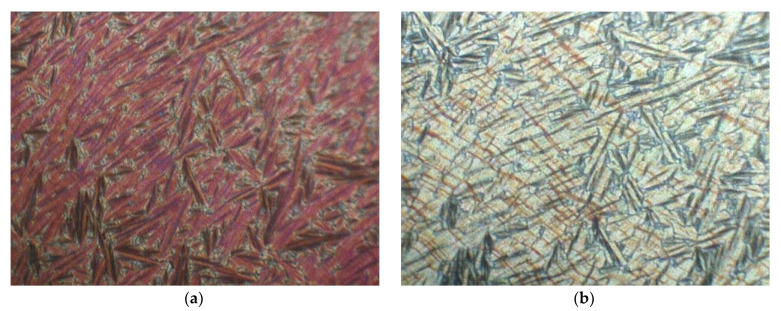

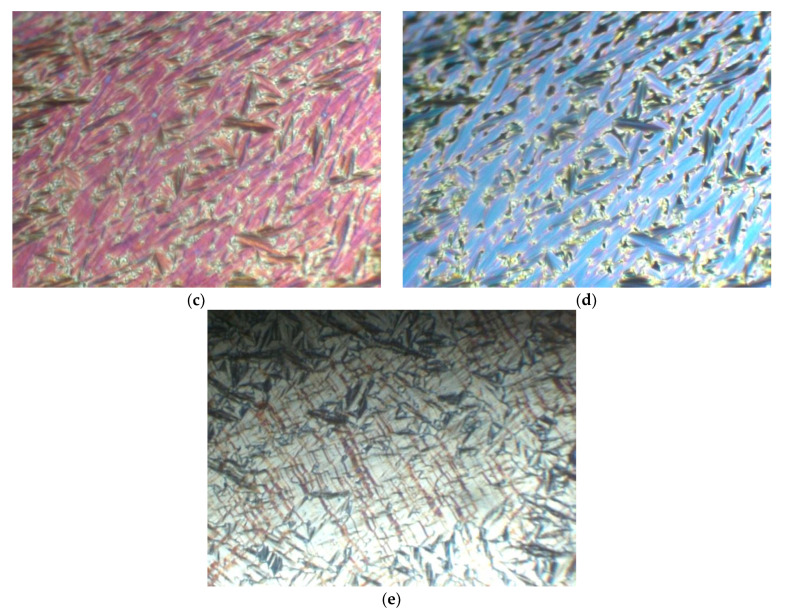
Optical textures under crossed polarizers in (**a**) SmC* phase (100 °C) for the Compound A+ Compound B system (x = 0.4), (**b**) SmC_A_*phase (85°C) phase for the Compound A + Compound B system (x = 0.4), (**c**) SmA* phase (107 °C) for the Compound C + Compound B system (x = 0.4), (**d**) SmC* phase (105 °C) for the Compound C + Compound B system (x = 0.4), (**e**) SmC_A_*phase (85 °C) phase for the Compound C + Compound B system (x = 0.4).

Figure 3a–e shows characteristic textures in the SmC_A_*, SmC* and SmA* phases exhibited by Compound A + Compound B system for molar concentration x = 0.4 and Compound C + Compound B system for molar concentration for x = 0.4.

### 3.2. Static Permittivity and Dielectric Anisotropy

**Figure 4:**
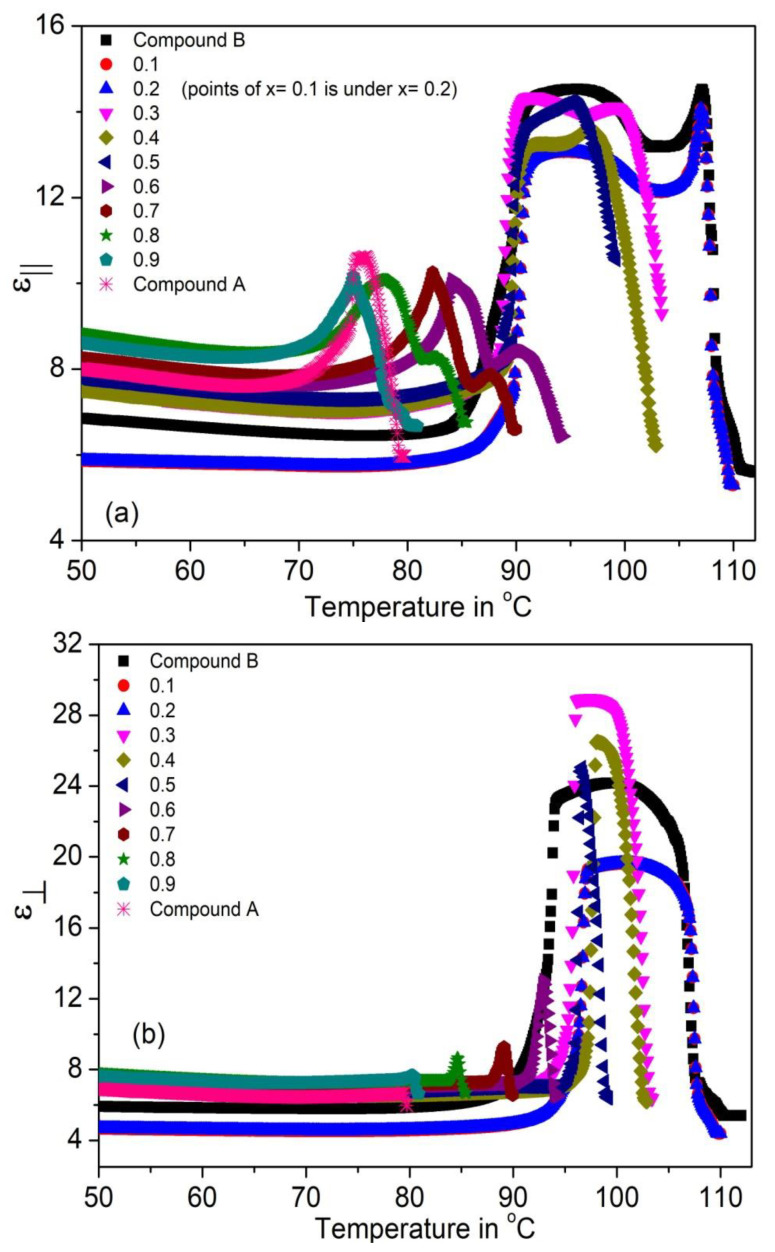

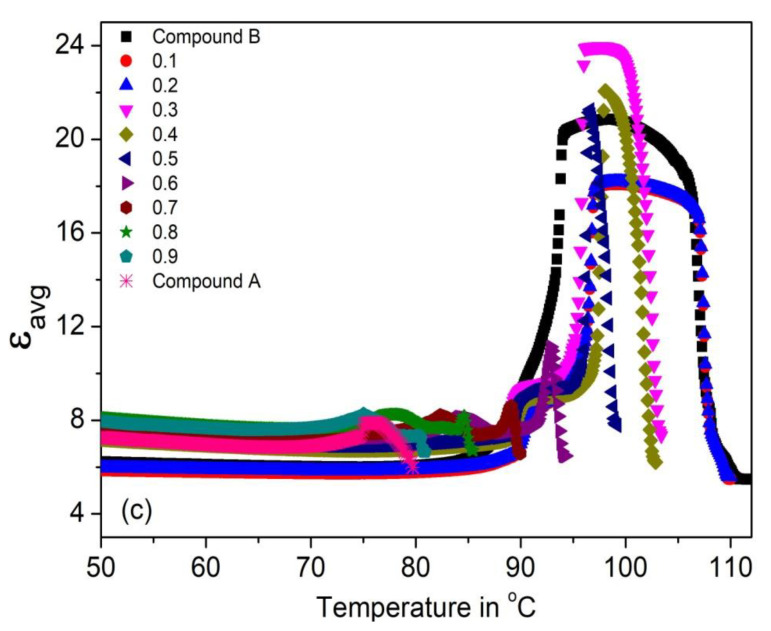
The (**a**) parallel, (**b**) perpendicular dielectric permittivity’s (ε_||_ and ε_⊥_) and (**c**) their average value (ε_avg_) of the Compound A + Compound B mixture system.

The temperature dependence of the parallel and perpendicular permittivity’s (ε_||_ and ε_⊥_) and their average value ε_avg_ = (2ε_⊥_ + ε_||_)/3 for the (Compound A + Compound B) system is shown in Figure 4a–c, respectively. Figure 4b shows that ε_⊥_ values peak in the SmC* phase (~19–28); this enhanced value is due to the temperature-dependent fluctuations of molecules in the direction of the phase angle of the helical structure [69,70,71,72,73,74,75]. The increment of ε_⊥_ is greater than ε_||_ in the SmC* phase. The values of ε_||_ and ε_⊥_ are small and almost constant in the SmC_A_* phase, it increases sharply at the SmC_A_* − SmC* phase transition temperature for all mixtures. The static permittivity’s contribution in the SmA* phase is weak and is mostly due to the amplitude fluctuation of the polarization vector [76]. Moreover, the absence of the SmC* phase in Compound A leads to its low permittivity value.

**Figure 5:**
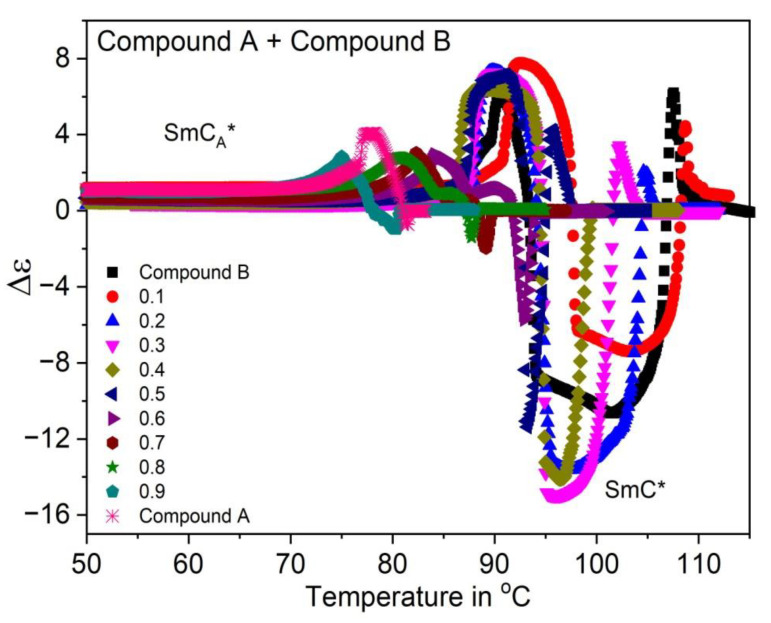
Temperature dependence of dielectric anisotropy (Δε) for the Compound A + Compound B mixture system. Negative dielectric anisotropy is observed in the SmC* phase for definite concentrations of Compound A in this mixture system.

The temperature dependence of the dielectric anisotropy (Δε) for Compound A + Compound B mixture system is shown in Figure 5. This system exhibits negative dielectric anisotropy in the SmC* phase as the increment of ε_⊥_ is more pronounced than that of ε_||_ near the vicinity of the SmA*-SmC* phase transition. The transverse dipole moment of the polar linking ester groups present in all the pure compounds considerably enhances ε_⊥_ with respect to the permittivity values ε_||_, along the molecular long axis. The dielectric anisotropy values however cross over to small positive values in the SmC_A_* phase. Additionally, as mentioned earlier, the dielectric anisotropy in the SmA^*^ phase is very small for all the mixtures.

**Figure 6:**
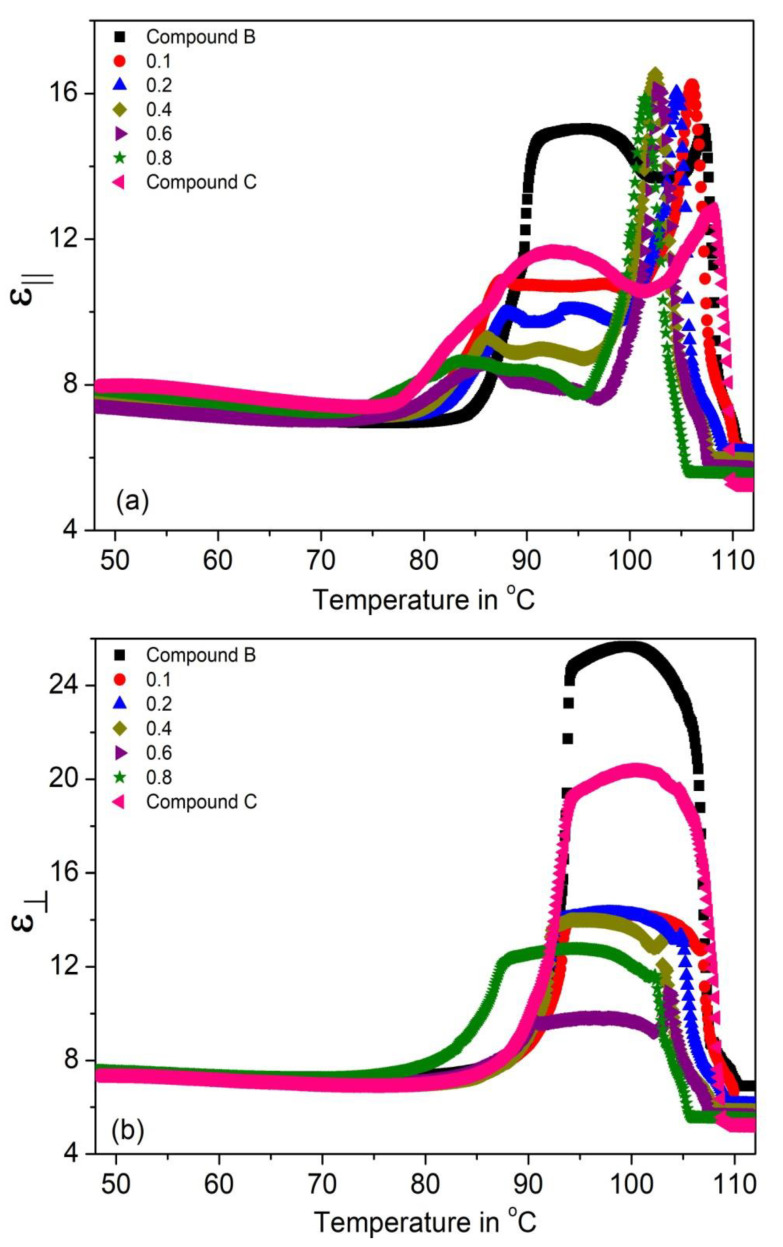

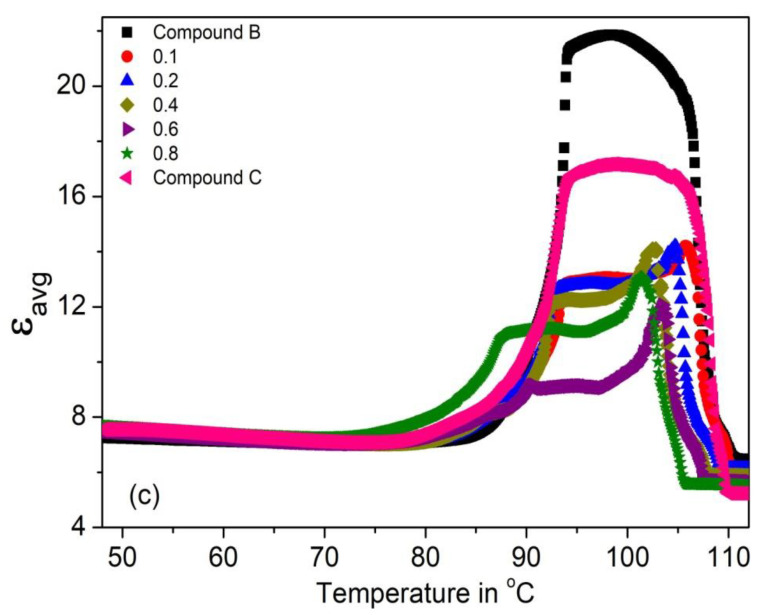
The (**a**) parallel, (**b**) perpendicular dielectric permittivity’s (ε_||_ and ε_⊥_) and (**c**) average value (ε_avg_) of the Compound C + Compound B mixture system.

The parallel and perpendicular (ε_||_ and ε_⊥_) static permittivity, and the average dielectric permittivity ε_avg_, respectively for Compound C + Compound B mixture system are shown in Figure 6a–c. The results show similar trends with respect to the Compound A + compound B system. It is observed that ε_||_ and ε_⊥_ increase in the SmC* phase following the Curie–Weiss law.

**Figure 7:**
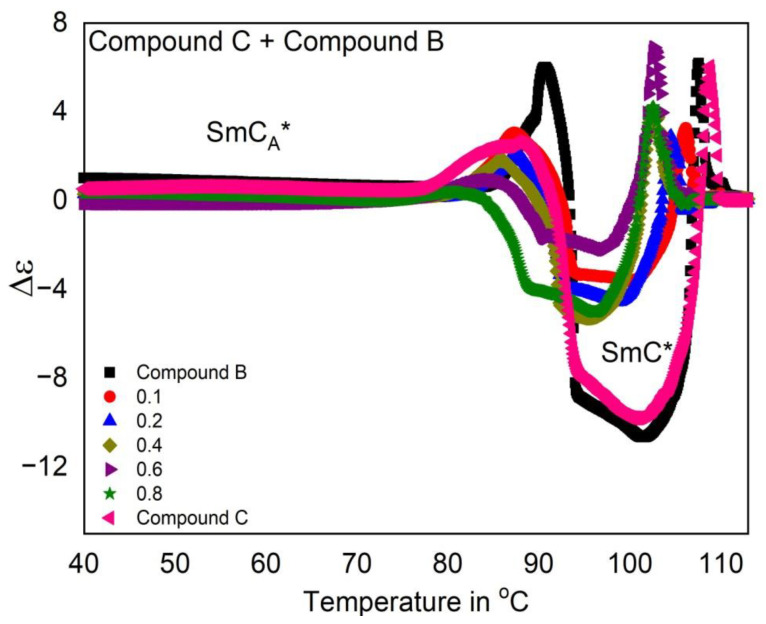
Temperature dependence of dielectric anisotropy (Δε) for the Compound C + Compound B mixture system.

As observed from Figure 7 for the Compound C + Compound B mixture system, Δε increases abruptly in the SmC* phase and becomes more and more negative with the SmC* phase stabilization (broader temperature range) on changing the concentration.

### 3.3. Helical Pitch

**Figure 8:**
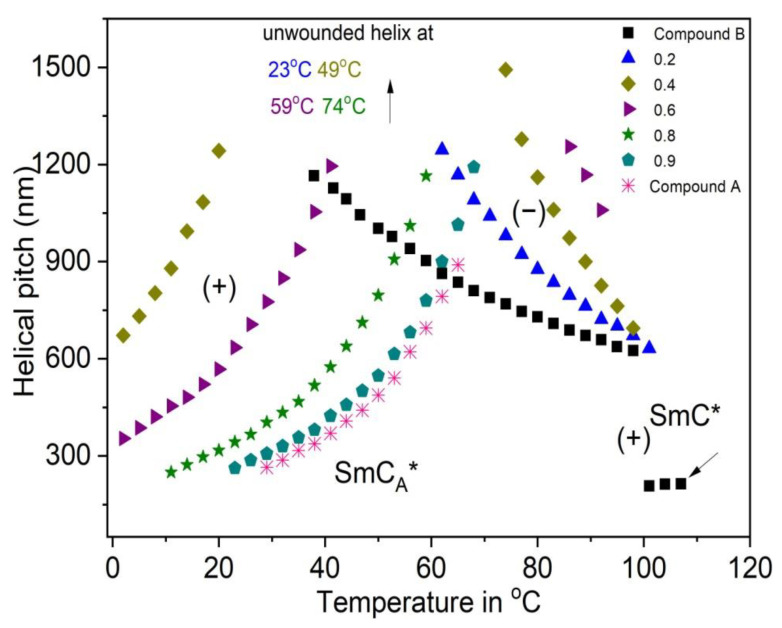
Temperature dependence of the helical pitch length for the Compound A + Compound B mixture system. Arrows of the corresponding color indicate the temperatures at which the helix became fully unwounded. The sense of the helical twist is indicated by “+” for the right-handed helix and by “− “for the left-handed helix.

**Figure 9:**
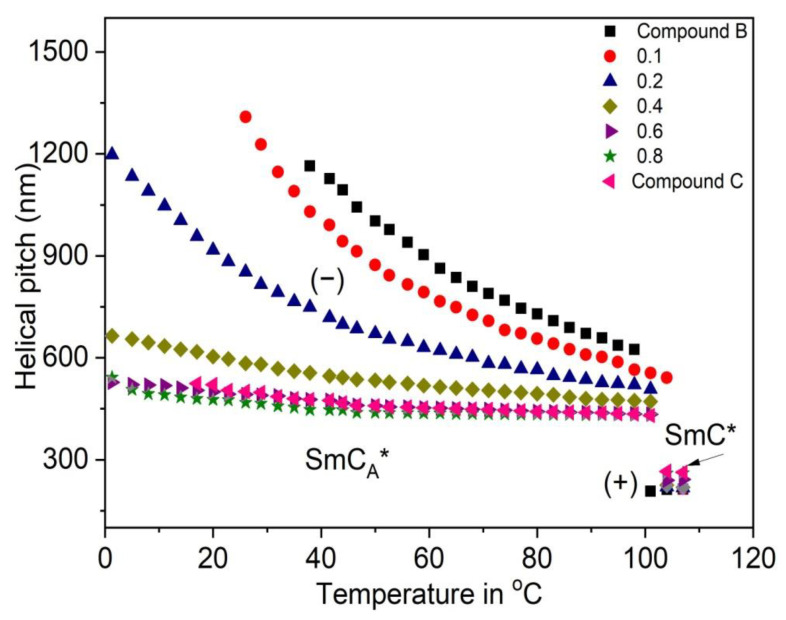
Temperature dependence of the helical pitch length for the Compound C + Compound B mixture system. The sense of the helical twist is indicated by “+” for the right-handed helix and by “− “ for the left-handed helix.

The results of the helical pitch measurements obtained for the Compound A + Compound B mixture system and Compound C + Compound B mixture system at different temperatures are presented in Figure 8 and Figure 9, respectively.

The sense of the helical twist (+ = right-handed helix, − = left-handed helix) and the temperature of the helix twist inversion (fully unwound helix) are also indicated in Figure 8. The parameters of the helix for chosen compositions of mixtures and pure compounds are compared. In the case of the Compound A + Compound B mixture system (for mixtures with x = 0.4 and x = 0.6), the right-handed helix at low temperatures and left-handed helix at high temperatures in the SmC_A_* phase is observed. For these mixtures, the helical twist sense inversion in the SmC_A_* phase is clearly present. In general, this behavior is typical for the system in which the compounds with different temperature characteristics and helix handedness are mixed [66]. Mixtures of the Compound C + Compound B mixture system form right-handed helix in the SmC* phase and left-handed helix in the SmC_A_* phase. As both compounds from this mixture system possess the same helix handedness in the SmC* as well as the SmC_A_* phases, the temperature characteristics of the helical pitch in binary mixtures are changed stepwise from one pure compound to the other. The helical pitch length measured in the SmC* phase is quite lower than that in the SmC_A_* phase.

**Figure 10:**
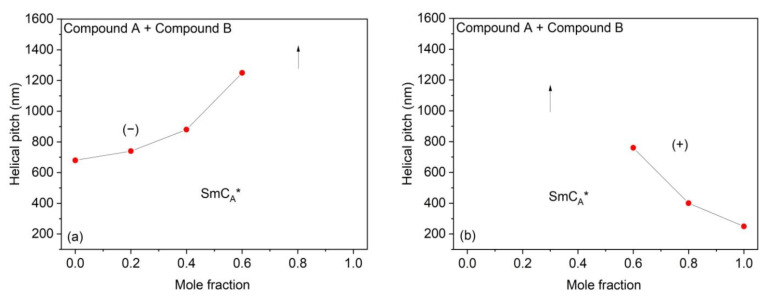
Dependence of helical pitch upon the concentration of compounds for the Compound A + Compound B mixture system at (**a**) 86 °C and (**b**) 29 °C. Arrows indicate concentration in which the helical pitch is above the measuring range of the spectrophotometer. The sense of the helical twist is indicated by “+”for the right-handed helix and by “− “for the left-handed helix.

Figure 10 shows the dependence of the helical pitch length upon the concentration of compounds for Compound A + Compound B mixture system at two selected temperatures: (a) 86 °C and (b) 29 °C. Arrows indicate concentration in which the helical pitch is above the measuring range of the spectrophotometer. The sense of the helical twist is indicated. The results show that for the appropriate mixture compositions, both at low and high temperatures, AFLC material with a very long helical pitch can be obtained.

**Figure 11:**
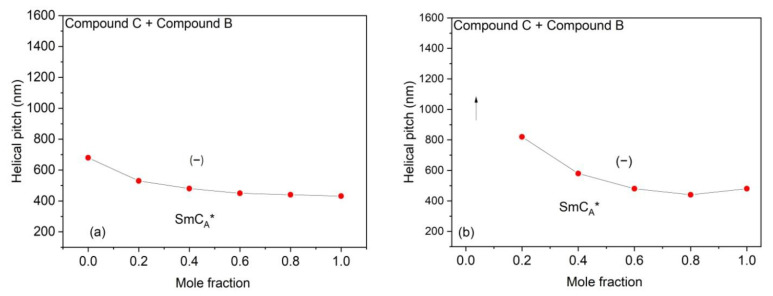
Dependence of helical pitch upon concentration of compounds for the Compound C + Compound B mixture system at (**a**) 86 °C and (**b**) 29 °C. Arrows indicate concentration in which the helical pitch is above the measuring range of the spectrophotometer. The sense of the helical twist is indicated by “− “for the left-handed helix.

Figure 11 shows the dependence of the helical pitch upon the concentration of the Compound C + Compound B mixture system at (a) 86 °C and (b) 29 °C. The mixtures exhibit a very short helical change in pitch in the SmC_A_* phase, regardless of the concentration and the temperature.

### 3.4. Spontaneous Polarization

**Figure 12:**
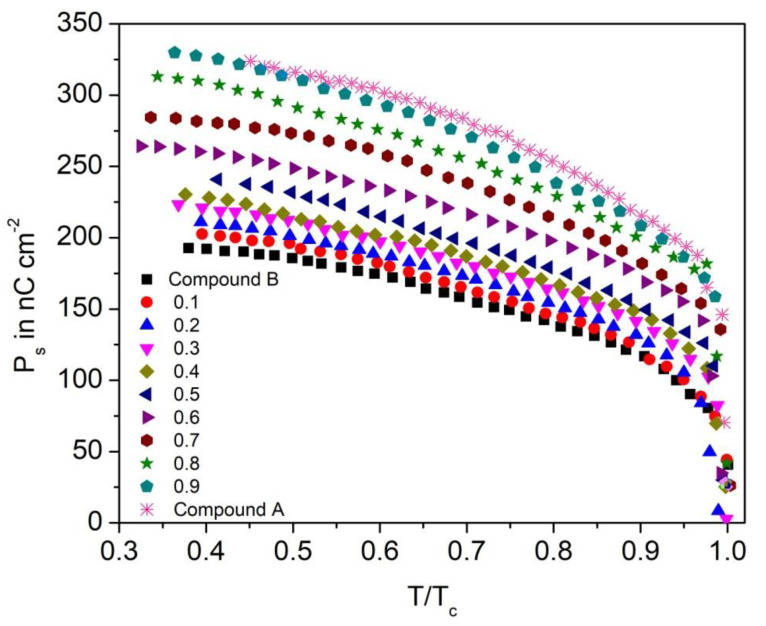
Experimental values of spontaneous polarization (P_s_) as a function of reduced temperature (T/T_c_) for the Compound A + Compound B mixture system.

Figure 12 displays the reduced temperature (T/T_C_) from Curie point T_C_ (SmA*-SmC* phase transition temperature) versus spontaneous polarization (P_s_) for the Compound A + Compound B mixture system at different concentrations. The continuity of the P_s_ curve implies that the nature of the SmC*-SmA* phase transition is of second order.

The value of P_s_ decreases with an increase in the temperature and attains their lowest values at the SmC*-SmA* transition temperature. Spontaneous polarization reaches ~340 nC.cm^−2^ in the SmC_A_* phase (30 °C); this high value of Ps is due to the contribution from the bulky nature of the chiral unit of the compounds, large dipole moment of the ester linkage and the high (43° to 45°) tilt angle of molecules with respect to the smectic layer normal, which increases the rotational hindrance barrier [67,69,77]. On comparing the variation of molar concentration with P_s_ of the Compound A + Compound B mixture system, P_s_ of Compound B is ~190 nC.cm^−2^ (mole concentration, x = 0.0) and it increases with the increase in molar concentration of Compound A in the mixture. Compound A has a propoxy-(CH_2_)_3_O- spacer group in the non-chiral part—the maximum values of P_s_ of Compound A is due to the volume effect, i.e., a propyl spacer occupies a greater volume than a pentyl spacer (Compound B) and by definition, P_s_ is proportional to the volume of that compound [77]. The Compound A + Compound B mixture system with mole fraction x = 0.1, 0.2, 0.3, 0.4, 0.5 possesses a comparatively low P_s_.

**Figure 13:**
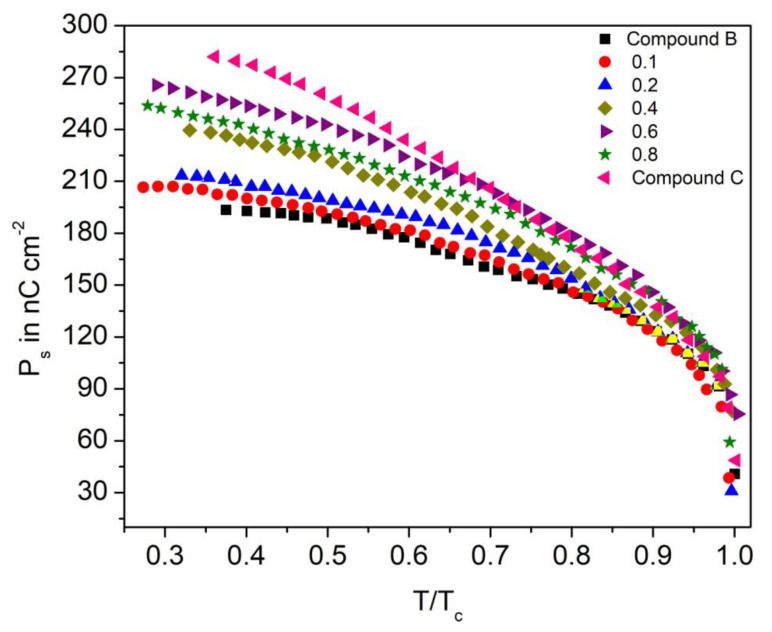
Experimental values of spontaneous polarization (P_s_) as a function of reduced temperature (T/T_c_) of the Compound C + Compound B mixture system.

The Compound C + Compound B mixture system possesses the P_s_ values in the range of 190–285 nC.cm^−2^ in the SmC_A_* phase, as shown in Figure 13. The P_s_ values are the minimum for Compound B, and they start to increase with the increase of the molar concentration of Compound C in the mixture. Maximum P_s_ of Compound C is caused by the non-fluorinated molecular core, whereas the low P_s_ of Compound B is due to the presence of one fluorine atom in the molecular core. This analysis is supported by the evidence that in laterally mono and di-fluorinated LC compounds, the orientation of the dipole moment of highly polar lateral fluorine atoms points in an opposite direction with respect to the orientation of the core and the chiral carbon atom. This fluorine atom in the molecular core of chiral molecules traps the π electrons and pulls them away from the conjugation along the main molecular axis. Therefore, lateral substitution by fluorine atoms has a considerable impact on the molecular dipoles related to the generation of the polarization in the ferroelectric and antiferroelectric LCs [67].

### 3.5. Relaxation Time

**Figure 14:**
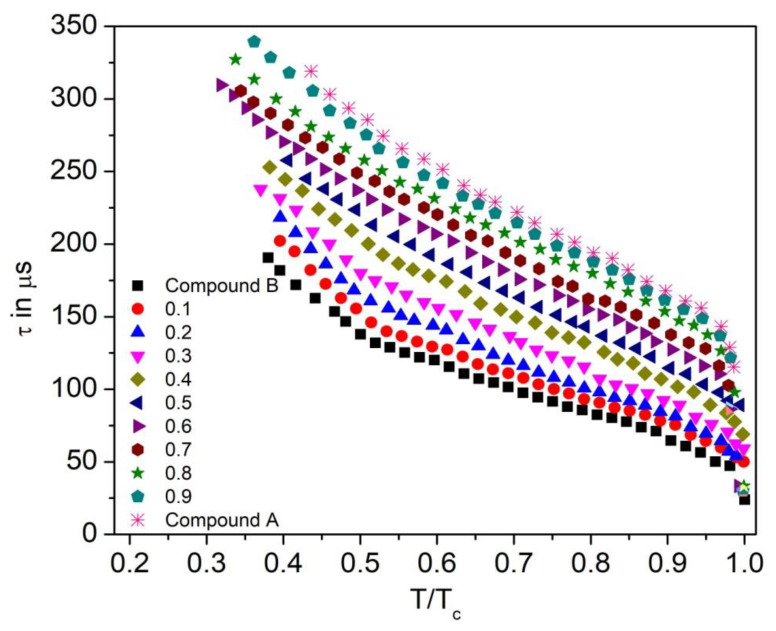
Relaxation time (τ) as a function of reduced temperature (T/T_c_) for the Compound A + Compound B mixture system.

Reduced temperature dependence of the relaxation time (τ) for the Compound A + Compound B mixture system is presented in Figure 14, where T_c_ is the SmA*-SmC phase transition temperature. The values of the relaxation time are below 340 μs in the measured range of the antiferroelectric phase of the mixtures. The relaxation time of the mixture is proportional to its viscosity.

The relaxation time of the mixture is proportional to its viscosity. With temperature increase, the viscosity of liquid crystal diminishes; therefore, the relaxation time also decreases. Relaxation time is maximum in the SmC_A_* phase due to the high tilt angle of molecules in this phase and is found comparatively less in the SmC* phase. Moreover, for this mixture system, mole fraction x = 0.1 has a lower relaxation time than x = 0.2, which denotes the rise in viscosity with an increase in the concentration of the mixture. Thus, the relaxation time increases with the increase in the concentration of Compound A in this mixture system.

**Figure 15:**
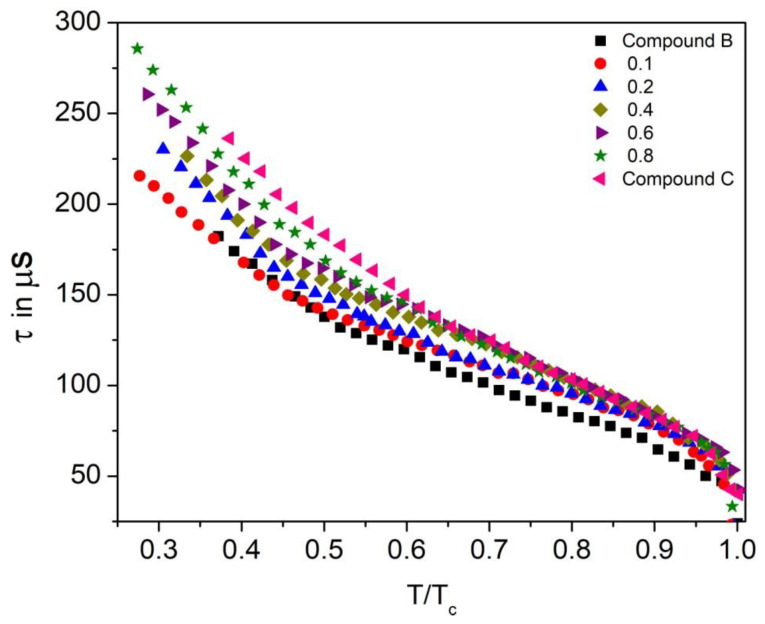
Relaxation time (τ) as a function of reduced temperature (T/T_c_) of the Compound C + Compound B mixture system.

The Compound C + Compound B mixture system has a relaxation time in the range of 185–275 μs in the SmC_A_^*^ phase, as shown in Figure 15. Similar to the Compound A + Compound B system, the relaxation time decreases with the increase in the concentration of Compound B in the mixtures. Low relaxation times, along with a broad range of the chiral smectic phase, are reported for this mixture system.

### 3.6. Effective Torsional Bulk Viscosity

**Figure 16:**
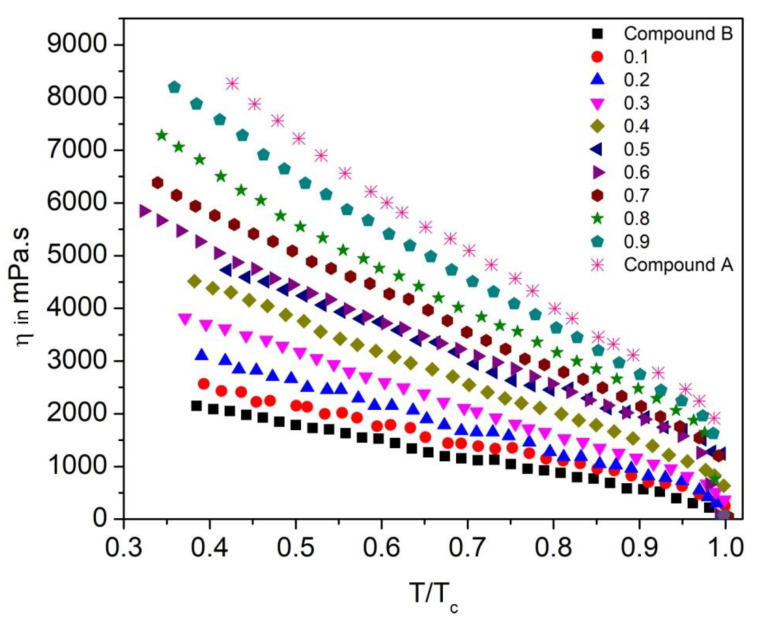
Effective torsional bulk viscosity (η) as a function of reduced temperature (T/T_c_) of the Compound A + Compound B mixture system.

To study the dynamic behavior of the FLC and AFLC liquid crystal system, it is important to investigate its effective torsional bulk viscosity (η) [67]. It signifies the speed of the molecular rotation for the SmC* cone upon switching under an applied electric field. Figure 16 shows η as a function of reduced temperature (T/T_c_) for the Compound A + Compound B mixture system. The η values are higher in the SmC_A_* phase than that in the SmC* phase and they follow almost similar trends, as it was observed for τ; η decreases with an increase in temperature. For this mixture system, η is minimum for pure Compound B (reaches ~2200 mPa.s in the SmC_A_* phase) and increases with the increase in molar concentration of Compound A in the mixture, reaching up to ~8000 mPa.s at 30 °C.

**Figure 17:**
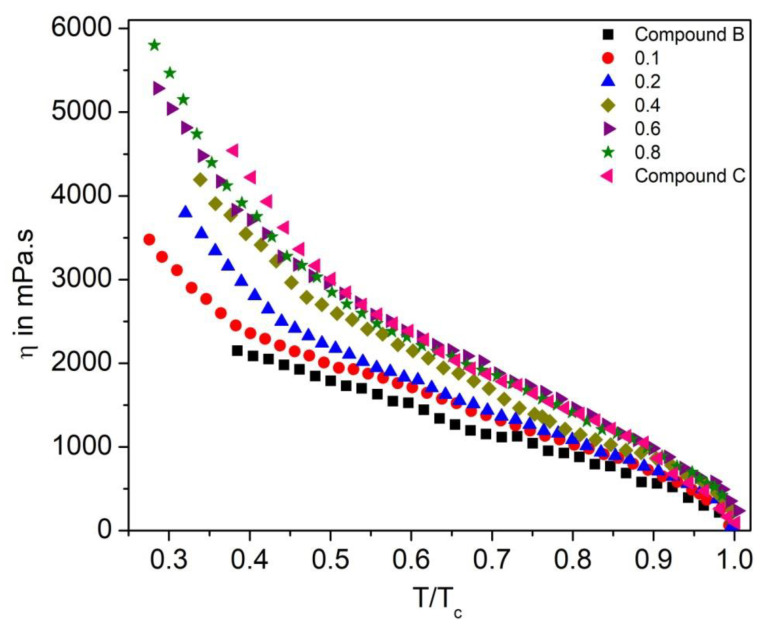
Effective torsional bulk viscosity (η) as a function of reduced temperature (T/T_c_) of the Compound C + Compound B mixture system.

Similar behavior is also observed for the Compound C + Compound B mixture system, as shown in Figure 17. As can be seen from Figure 17, η values are higher in the SmC_A_* phase as compared to that in the SmC* phase; η values decrease with the increase in molar concentration of Compound B in the mixture. In fact, LC materials with a low viscosity are suitable for the design of high-speed applications, such as virtual reality 3D video generations and sensing devices; therefore, mixtures with a molar concentration of up to x = 0.4 are potentially very promising candidates from a high-speed device application point of view.

### 3.7. Anchoring Energy Coefficients

The properties of molecular alignment, as well as the memory effect, have been greatly influenced by the anchoring strength of the molecules. This coefficient measures the deviation of the FLC molecule orientation from the anchoring direction. Two main anchoring strengths, that play an important role in ferroelectric and antiferroelectric liquid crystals, are the polarization and dispersion anchoring energy coefficients. FLCs phases have permanent dipole moments at the molecular level; the electrostatic force between the dipole moment of the surface and the liquid crystal molecules gives polarization anchoring strength (W_p_). On the contrary, dispersion anchoring strength (W_d_) may arise due to dispersion or van der Waals forces, i.e., the non-electrostatic interaction between the surface and the liquid crystal molecules [77,78,79,80].

#### 3.7.1. Dispersion Anchoring Energy Coefficient

**Figure 18:**
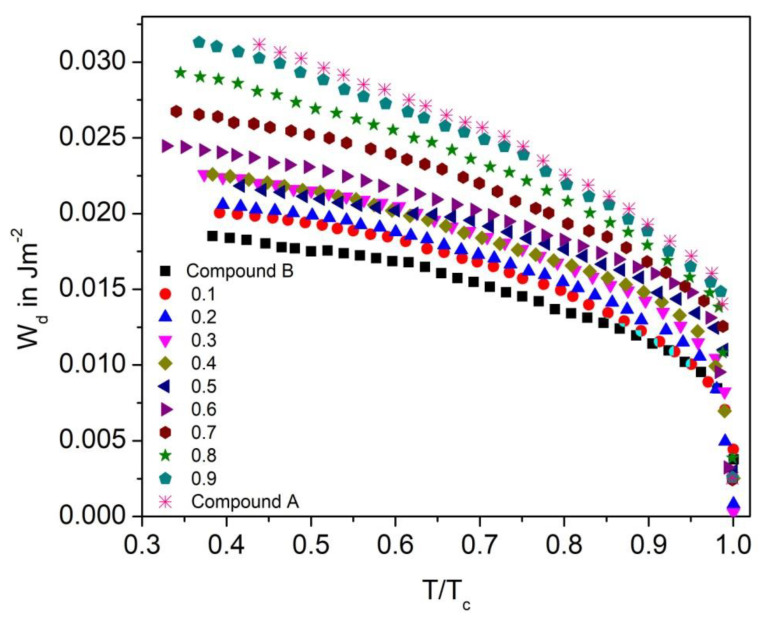
Dispersion anchoring energy coefficient (W_d_) versus reduced temperature T/T_C_ for the Compound A + Compound B mixture system.

Figure 18 shows the dispersion anchoring energy coefficient as a function of reduced temperature (T/T_C_) for the Compound A + Compound B mixture system. On increasing the temperature, the W_d_ values decrease and attain a minimum at the SmC*-SmA* phase transition temperature (Curie temperature). This pattern suggests that the enthalpy of the molecule continuously increased with temperature and thus broke their interaction barrier. The maximum value of W_d_ obtained in the Compound A + Compound B mixture is ~0.032 J.m^−2^ for Compound A and decreases with an increase in concentration of Compound B. For these binary mixtures, quite a small W_d_ value indicates the requirement of lower threshold voltage for the operation; therefore, the mixture with lower molecular concentration requires a low electric field to switch the molecules between the OFF and ON states.

**Figure 19:**
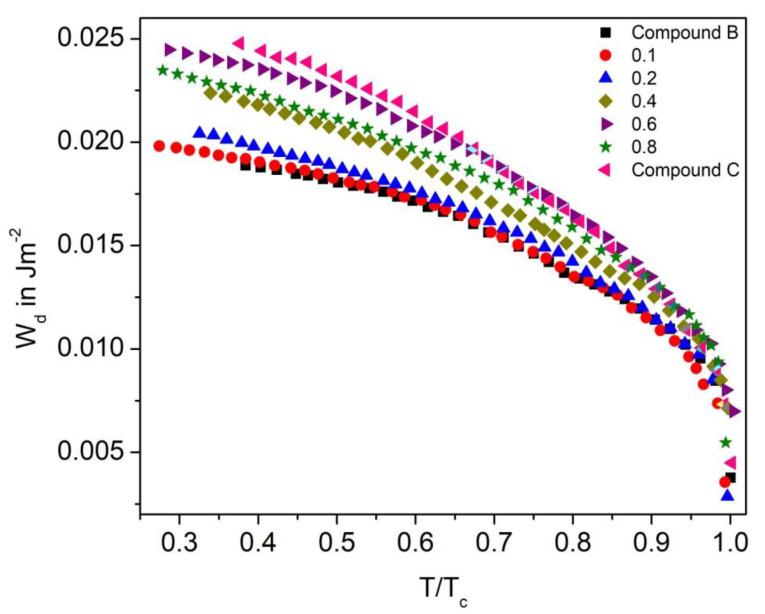
Dispersion anchoring energy coefficient (W_d_) versus reduced temperature T/T_C_ for the Compound C + Compound B mixture system.

The dispersion anchoring energy coefficient for the Compound C + Compound B mixture system also increases stepwise with the increase in molar concentration; however, its very high values are not preferable for practical applications. The maximum value of W_d_ in the Compound C + Compound B binary mixtures is ~0.025 J.m^−2^ for Compound C in the SmC_A_* phase, and similar to the Compound A + Compound B system, it decreases with an increase in mole fraction of Compound B (Figure 19).

#### 3.7.2. Polarization Anchoring Energy Coefficient

**Figure 20:**
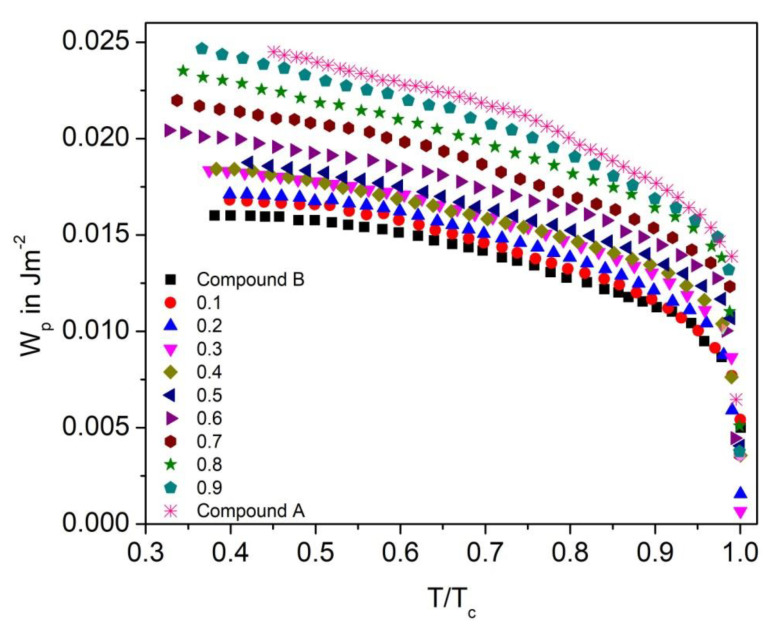
Polarization anchoring energy coefficient (W_p_) versus reduced temperature T/T_C_ for the Compound A + Compound B mixture system.

**Figure 21:**
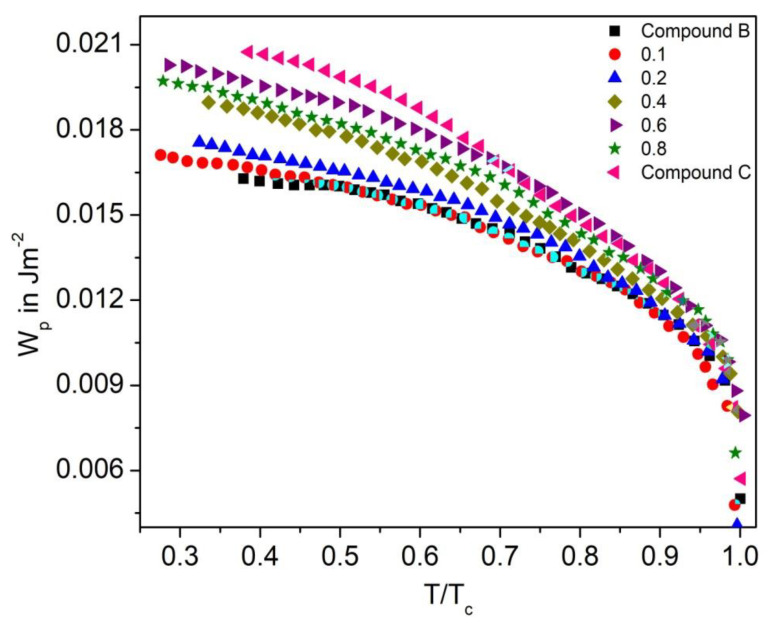
Polarization anchoring energy coefficient (Wp) versus reduced temperature T/T_C_ for the Compound C + Compound B mixture system.

The polarization anchoring energy coefficient (W_p_) for the Compound A + Compound B and Compound C + Compound B mixture system is shown in Figure 20 and Figure 21, respectively. Characteristic decreasing of W_p_ with increasing temperature on approaching the SmC*-SmA* phase transition is found for both the mixture systems. The low value of W_p_ and W_d_ for the binary mixtures holds under the condition that P_s_ and viscosity are low.

A comparative result of a study of the electro-optical properties for the Compound A + Compound B mixture system and Compound C + Compound B mixture system extrapolated to 20 °C, are shown in Table 1 and Table 2, respectively. Mixtures with mole fraction, x = 0.2, 0.3, 0.4, 0.5 and 0.6 of the Compound A + Compound B system shows promising material properties due to their relatively high P_s_ values, fast switching times and moderate values of rotational viscosity along with a wide-ranged SmC_A_* phase. Similarly, for the Compound C + Compound B mixtures, the appropriate mole fractions for advanced exploration are x = 0.2, 0.4 and 0.6. Such materials, therefore, may be used as starting materials to dope with monomers and dimers to investigate the changes in their physical parameters.

## 4. Conclusions

Two newly formulated bi-component orthoconic antiferroelectric liquid crystalline mixture systems were designed and their basic parameters were investigated. The temperature and the mole fraction dependence of helical pitch, static dielectric permittivity, dielectric anisotropy and electro-optical properties of the Compound A + Compound B and Compound C + Compound B mixture system have been studied.

Depending on the concentration of the components, the investigated mixture exhibit paraelectric (SmA*), ferroelectric (SmC*) and antiferroelectric (SmC_A_*) phases over a reasonably broad temperature range down to room temperatures. In the Compound A + Compound B system, a gradual enhancement of the SmC_A_* phase is observed. However, within this system, for mixtures having a direct phase transition from the isotropic phase to the SmC_A_* phase, the range of the SmC_A_* phase increases up to 12.5 °C at x = 0.6, which can potentially be utilized in OAFLC mixtures due to their inherent chevron defect elimination properties. An enhancement in the temperature range of the SmC_A_* phase is also noticed in the Compound C + Compound B system.

Both designed mixture systems exhibit sufficiently large values of spontaneous polarization (P_s_ above 200 nC.cm^−2^), negative dielectric anisotropy, fast switching speed (τ below 350 µs), and low viscosity (below 8 Pa.s) at room temperature. These mixtures can be further polymer-stabilized and used as an attractive candidate for mixtures in high-speed sensing applications.

The dispersion anchoring energy coefficients values range from 0.013 to 0.035 Jm^−2^; such a lower value indicates the requirement of lower threshold voltage to switch the molecule between the OFF and ON state, which is an important property for the design of effective switching devices.

Mixtures with optimal electro-optic properties from both the binary systems under investigation have been identified. They are x = 0.2, 0.3, 0.4, 0.5 and 0.6 of the Compound A + Compound B mixture system, and x = 0.2, 0.4 and 0.6 for the Compound C + Compound B mixture system. It is proposed that these mixtures will be further polymer-stabilized by adding suitable reactive monomers and dimers to stabilize the structure and properties of the antiferroelectric state in order to symmetrise the performance of surface-stabilized and deformed-helix effects. Further studies in this direction are in progress and will be published separately elsewhere.

## Figures and Tables

**Table 1:** Electro-optical properties of the Compound A + Compound B mixture system extrapolated to 20 °C.

Mole Fraction (x)	P_s_ (nC·cm^−2^)	τ (µs)	ƞ (mPa.s)	W_d_ (J·m^−2^)	W_p_ (J·m^−2^)
0.0	199	256	2600	0.020	0.016
0.1	213	261	3568	0.021	0.017
0.2	222	299	3567	0.022	0.017
0.3	235	285	4548	0.024	0.019
0.4	244	314	5400	0.024	0.020
0.5	254	336	5577	0.025	0.020
0.6	266	377	7186	0.025	0.021
0.7	288	379	8090	0.028	0.024
0.8	323	415	8945	0.031	0.026
0.9	345	413	10102	0.034	0.027
1.0	347	419	10554	0.034	0.028

**Table 2:** Electro-optical properties of the Compound C + Compound B mixture system extrapolated to 20 °C.

Mole Fraction (x)	P_s_ (nC.cm^−2^)	τ (µs)	ƞ (mPa.s)	W_d_ (J.m^−2^)	W_p_ (J.m^−2^)
0.0	199	256	2600	0.019	0.016
0.1	202	273	5470	0.020	0.018
0.2	217	297	5653	0.021	0.018
0.4	246	313	6573	0.024	0.020
0.6	279	340	7529	0.026	0.021
0.8	266	374	8400	0.025	0.020
1.0	294	356	6639	0.027	0.021

## Data Availability

Excluded.
